# iTRAQ-Based Proteomics Analysis of Plasma of Myasthenia Gravis Patients Treated with Jia Wei Bu Zhong Yi Qi Decoction

**DOI:** 10.1155/2019/9147072

**Published:** 2019-12-13

**Authors:** Yunke Zhang, Junhong Yang, Yingzhe Chen, Jie Lv, Jing Zhang, Yingna Zhang, Xue Zhao, Hua Fang, Chongchong Liu, Qingyong Zhang, Xinzheng Cui, Xiaohan Wang, Feng Gao

**Affiliations:** ^1^School of Rehabilitation Medicine, Henan University of Chinese Medicine, No. 156 Jinshui East Road, Zhengzhou City, Henan Province 450008, China; ^2^Department of Neurology, The First Affiliated Hospital of Henan University of Chinese Medicine, No. 19, Renmin Road, Zhengzhou City, Henan Province 450000, China; ^3^Department of Neurology, Pingdingshan Traditional Chinese Medicine Hospital, Henan No. 4 Courtyard, North Section of Zhongxing Road, Pingdingshan City 467000, China; ^4^Department of Neuroimmunology Research, Henan Institute of Medical and Pharmaceutical Sciences, Zhengzhou University, Henan, No. 40, University Road, Zhengzhou City, Henan Province 450052, China; ^5^Beijing University of Chinese Medicine, No. 11, North Third Ring Road, Chaoyang District, Beijing 100029, China; ^6^Myasthenia Gravis Comprehensive Diagnosis and Treatment Center, Henan Provincial People's Hospital, No. 7, Weiwu Road, Zhengzhou City, Henan Province 450003, China; ^7^Henan University of Chinese Medicine, No. 156 Jinshui East Road, Zhengzhou City, Henan Province 450008, China

## Abstract

Myasthenia gravis (MG) is an autoimmune disease. A proportion of MG patients did not get satisfactory results after treatment with pyridostigmine and prednisone. Jia Wei Bu Zhong Yi Qi (Jia Wei BZYQ) decoction, a water extract from multiple herbs, has been demonstrated to be effective in the treatment of multiple “Qi deficiency type” diseases including MG in China. In this text, we investigated protein alterations in the plasma from healthy volunteers (C), MG patients without any treatment (T1), MG patients with routine western medical treatment (T2), and MG patients with combined treatments of Jia Wei BZYQ decoction and routine western medicines (T3) and identified some potential proteins involved in the pathogenesis and treatment of MG. iTRAQ (isobaric tags for relative and absolute quantitation) and 2D-LC-MS/MS (two-dimensional liquid chromatography-tandem mass spectrometry technologies) were employed to screen differentially expressed proteins. The identification, quantification, functional annotation, and interaction of proteins were analyzed by matching software and databases. In our project, 618 proteins were identified, among which 447 proteins had quantitative data. The number of differentially expressed proteins was 110, 117, 143, 115, 86, and 158 in T1 vs. C, T2 vs. C, T2 vs. T1, T3 vs. C, T3 vs. T1, and T3 vs. T2 groups, respectively. Functional annotation results showed that many differentially expressed proteins were closely associated with immune responses. For instance, some key proteins such as C-reactive protein, apolipoprotein C-III, apolipoprotein A-II, alpha-actinin-1, and thrombospondin-1 have been found to be abnormally expressed in T3 group compared to T1 group or T2 group. Interaction network analyses also provided some potential biomarkers or targets for MG management.

## 1. Introduction

Myasthenia gravis (MG) is a disorder of neuromuscular transmission with an incidence of 0.3 to 2.8 cases per 100,000 people and an annual mortality of 0.06 to 0.89 per million people worldwide [1–3]. MG patients can generate autoantibodies against postsynaptic neuromuscular proteins and epitopes such as acetylcholine receptor (AChR), muscle-specific tyrosine kinase (MuSK), and lipoprotein receptor-related protein-4 (LRP4) to attack the body's tissues [4–6]. MG with autoantibodies against AChR (AChR-MG) is the most common MG subtype, accounting for about 70%–80% of all MG cases [7]. MuSK antibodies are found in 1–10% of MG patients, and LRP4 antibodies can be detected in approximately 7% of MG patients without antibodies against AChR and MuSK [8]. AChR antibodies mainly occur in generalized and ocular MG (both early-onset and late-onset) with thymic hyperplasia as the common feature of early-onset MG and atrophic thymus and fat tissue-replaced thymus as the frequent pathological manifestations of late-onset MG [8]. Moreover, AChR antibodies are common in patients with MG and thymoma [4]. The concentration of total AChR antibody was not directly related to MG severity, whereas AChR antibody concentration is increased when the condition for MG patients is exacerbated [7, 8]. MG patients with AChR or MuSK antibodies usually develop more severe symptoms (51-52% MGFA I-II at onset) compared with LRP4 antibody-positive subgroup [7–9]. Moreover, MG patients with double-positive autoantibodies of AChR/LRP4 or MuSK/LRP4 have more severe symptoms relative to any single-positive MG subgroup [9]. It is presumed that thymus is not related to the pathogenesis of MG in MG patients with MuSK antibodies, and extremely rare MuSK antibodies are found in MG patients with thymoma [4]. MG patients with positive LRP4 antibodies usually have ocular or mild generalized symptoms (85% with MGFA grade I or II at disease onset), and some have thymic changes (31% hyperplasia, 29% involuted thymus, 7% atrophy, 33% normal thymus, and none with thymoma) [9]. The average age for MG patients is 33.4 years for females and 41.9 years for males at disease onset [9].

MG is characterized by multiple clinical symptoms such as muscle weakness, drooping eyelids, double vision, trouble talking, and trouble walking [10, 11]. Most MG patients have good prognosis due to great advances in diagnostic techniques, epidemiologic methodologies, and treatment for MG over the past several decades [2, 10, 12]. However, only a few MG patients have complete remission and most patients need sustained treatment to alleviate related symptoms [13]. Moreover, MG diagnosis is often different at the beginning of disease due to its heterogeneity [14]. Hence, it is imperative to explore MG pathogenesis and search for potential biomarkers or targets for MG management.

Recently, mass spectrometry- (MS-) based iTRAQ (isobaric tag for relative and absolute quantification) technique has become prominent in proteomics research requiring relative quantification [15, 16]. Emerging study shows that iTRAQ is an effective chemical tagging strategy that offers a deep insight into the molecular mechanisms implicated in disease progression and drug responses [16, 17]. For instance, iTRAQ-based quantitative proteomic analyses identified key proteomic changes and critical metabolic pathways in arsenic-induced liver fibrosis rat models [18]. Additionally, Wang et al. pointed out that iTRAQ-based proteomic analysis may reveal the molecular targets of drugs and bioactive small molecules [19].

Immunotherapy with glucocorticosteroids and symptomatic treatments with acetylcholinesterase inhibitors are the bedrock for MG management [2, 12]. Pyridostigmine and prednisone have been widely used as the first-line therapeutic drugs for MG patients [2]. However, the uneven absorption and side effects of pyridostigmine and prednisone limited their clinical applications [2]. Traditional Chinese medicine (TCM) is a holistic medical system for diagnosis, prevention, and treatment of diseases and has been an integral part of Asian cultures for thousands of years [20]. Some studies pointed out that combined therapy of TCM and western medicines could markedly improve clinical outcomes of MG patients with reduced side effects [21, 22].

Bu Zhong Yi Qi (BZYQ) decoction (also called “Bojungikki-tang” or “Hochu-ekki-to”), a water extract from multiple herbs, has been widely used to treat “Qi deficiency type” or “Yang deficiency type” diseases in Asia [23, 24]. BZYQ decoction can enhance immunological responses, improve nutritional status, ameliorate chronic fatigue syndromes, and reduce cytotoxicity of chemotherapeutic drugs [25–28]. BZYQ decoction has therapeutic effects on multiple diseases such as tumors [29, 30], chronic obstructive pulmonary disease [25], and MG [31]. In this study, two herbs (*Curculigo orchioides* Gaertn and *Epimedium baiealiguizhouense* S.Z.He & Y.K.Yang) that can ameliorate kidney-Yang deficiency syndromes and replenish kidney essence were added into the formula of BZYQ decoction to generate Jia Wei BZYQ decoction [32, 33]. Clinical studies over many years in China presented that Jia Wei BZYQ decoction was effective and safe to treat MG [34, 35]. Moreover, our antecedent finding revealed that Jia Wei BZYQ decoction could markedly decrease acetylcholine receptor antibody (AChR-Ab) serum level and reduce concentrations of IL-2, IL-6, IL-17A, and IFN-*γ* in thymus and spleen tissue fluid in experimental autoimmune MG rat models [36]. Also, our researchers found that Jia Wei BZYQ decoction in combination with pyridostigmine bromide was more effective to alleviate the clinical symptoms, reduce serum AChR-Ab level, and decrease Th17 cell proportion without obvious side effects compared with pyridostigmine bromide alone in the treatment of MG patients with spleen and kidney deficiency syndromes [37, 38]. However, the pharmacological basis for Jia Wei BZYQ decoction in the treatment of MG has not been well defined at present.

In the present study, iTRAQ and 2D-LC-MS/MS technologies as well as bioinformatics approaches were used to investigate potential plasma biomarkers in MG patients treated with routine western medicines (prednisone and/or pyridostigmine) alone or along with Jia Wei BZYQ decoction. In addition, we identify some vital proteins associated with MG etiology.

## 2. Methods

### 2.1. Clinical Information

Blood samples were collected from healthy volunteers (*n* = 3, C group, labeled 113, 18–70 years old) and ambulant or hospitalized primary MG patients (*n* = 9, 18–70 years old) at the First Affiliated Hospital of Henan University of Traditional Chinese Medicine and the affiliated hospital of Henan Medical Science Research Institute between April 2016 and January 2017. MG patients were diagnosed according to western medicine criteria as previously described [39]. MG severity was assessed following the modified Osserman classification standard as described in a previous document [39]. MG patients with a stable disease status and a modified Osserman I or IIA subtype were enrolled in our project. In addition, MG patients need to have main symptoms and at least one minor symptom of spleen and kidney deficiency based on TCM diagnostic criteria. Main symptoms include (i) ptosis or diplopia; (ii) articulation, chewing and swallowing difficulties, and choking and coughing while drinking water; and (iii) fatigability of the whole body. Minor symptoms contain some features of Qi deficiency and Yang deficiency. Qi deficiency is characterized by shortness of breath, sluggish eyes, complexion chlorosis, body fatigue, loss of control over bowel movements, light or dark red tongue with a thin white coating, and a weak pulse. The characteristics of Yang deficiency are cold limbs, fear of cold, abdominal pain, borborygmus, light or dark red tongue with a thin white coating, and a weak pulse. Moreover, MG patients with other diseases, patients underwent thymectomy or plasma exchange therapy, and patients in prenatal and suckling periods were excluded from our study. All participants signed written informed consent documents, and our study was approved by the Ethical Committee of the First Affiliated Hospital of Henan University of Traditional Chinese Medicine.

Nine MG patients were divided into 3 groups as follows: untreated group (*n* = 3, T1, labeled 114), a routine western medicine treatment group (*n* = 3, T2, labeled 115), and combined treatment group of routine western medicine plus Jia Wei BZYQ decoction (*n* = 3, T3, labeled 116). MG patients in T2 group were treated with prednisone or pyridostigmine bromide tablets, alone or in combination. At the beginning, 60–80 mg prednisone was administered once daily to MG patients for 20 days; the dose was gradually decreased at a rate of 2.5 mg/15 days according to the status of patients. Pyridostigmine bromide tablets were given to MG patients at a dosage of 60 mg × 4 times/day for 20 days; the dose was gradually reduced by 5 mg/15 days according to the status of patients. MG patients in T3 group were given Jia Wei BZYQ decoction orally for 2 months with twice daily (1 pack each time) on the basis of the routine western medicine treatment as T2 group.

Jia Wei BZYQ decoction was generated by the First Affiliated Hospital of Henan University of Chinese Medicine from a boiled water extraction of 10 traditional Chinese herbal medicines with the prescription as below: *Astragalus membranaceus* (Fisch.) Bunge (50 g, Lot: 161101QF), *Glycyrrhiza uralensis* Fisch. (15 g, Lot: 160829), *Bupleurum chinense* DC. (15 g, Lot: 160601QF), *Angelica sinensis* (Oliv.) Diels (10 g, Lot: 160402QF), *Codonopsis pilosula* (Franch.) Nannf. (25 g, Lot: 161202), *Atractylodes macrocephala* Koidz. (15 g, Lot: 160701QF), *Cimicifuga foetida* L. (10 g, Lot: 160601), *Citrus reticulata* Blanco peel (10 g, Lot: 160101), *Curculigo orchioides* Gaertn. (15 g, Lot: 16101910), and *Epimedium baiealiguizhouense* S.Z.He & Y.K.Yang (15 g, Lot: 161101QF). All raw herbs were purchased from Zhang Zhongjing Pharmacy (Zhengzhou, China). For extraction of Jia Wei BZYQ decoction, 10 herbal medicines were boiled in water for a total of 4 hours with water. The extraction step was repeated once. Then, the two extracts were mixed and subpackaged into bags with 200 ml in each pack. Basic clinical parameters of healthy volunteers and MG patients before treatment are displayed in Table 1. Clinical features of MG patients before and after treatment along with related treatment methods and clinical outcomes are displayed in Table 2.

### 2.2. Blood Sample Collection

Blood samples (10 ml) were collected using EDTAK2 anticoagulation tubes on the second day after enrolling and two months later after treatment and then centrifuged at 4000 r/min for 5 min at room temperature. Then, the plasma in the supernatants was collected and subpackaged in freezer tubes and stored at liquid nitrogen.

### 2.3. iTRAQ 2D-LC-MS/MS-Based Proteomic Analysis

Plasma samples were treated with 200 *μ*l triethylammonium bicarbonate (TEAB) solution and then subjected to ultrasonication and high-speed centrifugation (12,000 r/min, 20 min). Next, cell supernatants were precipitated using 4 volume exchanges of ice-cold acetone containing 10 mM dithiothreitol (DTT). After 2 h of incubation, cell precipitation was collected by centrifugation (12000 r/min, 20 min) and then resuspended in 800 *μ*l ice-cold acetone containing 10 mM DTT. Next, protein precipitation was collected by high-speed centrifugation (12,000 r/min for 20 min), air-dried, and dissolved in 100 *μ*l TEAB buffer. Protein concentration was determined by a Bradford microassay (Bio-Rad Laboratories, Hercules, CA, USA) with BSA as the standard as previously described [40, 41]. Then, protein (100 *μ*g/100 *μ*l) was diluted using 500 *μ*l NH_4_HCO_3_ (50 mM) and digested overnight at 37°C with 2 *μ*g trypsin solution, followed by acidification using an equal volume of 0.1% fatty acid (FA). Next, the acidified solution was poured through a methanol-activated and 0.1% FA-balanced Strata-X C18 column 3 times. After washed twice with 0.1% FA + 5% acetonitrile, the column was eluted once with 1 ml 0.1% FA + 80% acetonitrile. Subsequently, 1 ml elution was freeze-dried and redissolved in TEAB solution. Next, the peptides were labeled with 8-plex iTRAQ reagents (SCIEX, Redwood City, CA, USA) following the protocols provided by the manufacturer. Next, samples were fractionated using a Durashell C18 column (5 *μ*m, 100 Å, 4.6 × 250 mm, Agela Technologies, Tianjin, China) on a Thermo DINOEX Ultimate 3000 BioRS system (Thermo Scientific, Waltham, MA, USA). Subsequently, the peptides were analyzed by tandem mass spectrometry (MS/MS) on an AB SCIEX NanoLC-MS/MS instrument (Triple TOF 5600 plus, SCIEX).

### 2.4. Database Searching and Protein Identification

MS data were analyzed via MASCOT and Protein Pilot software to identify and quantify corresponding proteins in different treatment groups. Interaction of identified proteins was analyzed using the STRING database. Functional annotations on identified proteins were performed by Gene Ontology (GO) database, Kyoto Encyclopedia of Genes and Genomes (KEGG) pathway database, and Clusters of Orthologous Groups of proteins (COGs) database.

### 2.5. Statistical Analysis

One-way analysis of variance (ANOVA) and Turkey's post hoc test were used to measure the difference of multiple groups with *P* < 0.05 as the threshold for statistical significance. Proteins were considered as differentially expressed when satisfying the following two conditions simultaneously: (a) *P* value <0.05 and (b) upregulated ratio ≥1.5 or downregulated ratio ≤0.67.

## 3. Results

### 3.1. Protein Identification from Plasma Samples

To screen MG-related protein markers and explore potential therapeutic mechanisms of Jia Wei BZYQ decoction and routine western medicines for MG, iTRAQ and 2D-LC-MS/MS technologies were used to analyze differentially expressed plasma proteins in MG patients treated with or without routine western medicines alone or along with Jia Wei BZYQ decoction. In our study, a total of 618 proteins were identified with 447 proteins having quantitative results. Quantitative results revealed that the number of differentially expressed proteins in the groups of T1 vs. C, T2 vs. C, T2 vs. T1, T3 vs. C, T3 vs. T1, and T3 vs. T2 was 110, 117, 143, 115, 86, and 158, respectively (Table 3). Additionally, 67, 53, 67, 64, 38, and 82 proteins were upregulated (ratio ≥ 1.5) and 43, 64, 76, 51, 48, and 76 proteins were downregulated (ratio ≤ 0.67) in the groups of T1 vs. C, T2 vs. C, T2 vs. T1, T3 vs. C, T3 vs. T1, and T3 vs. T2, respectively (Table 3). Moreover, the names and fold changes of differentially expressed proteins in T1 vs. C and T3 vs. T2 groups are presented in a heat map (Supplementary Figures [Supplementary-material supplementary-material-1] and [Supplementary-material supplementary-material-1]).

### 3.2. Functional Annotation of Identified Proteins

**Figure 1:**
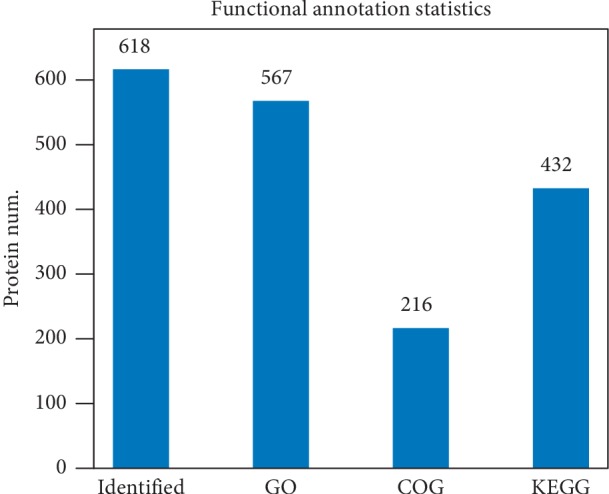
The number of proteins with functional annotation in different databases.

**Figure 2:**
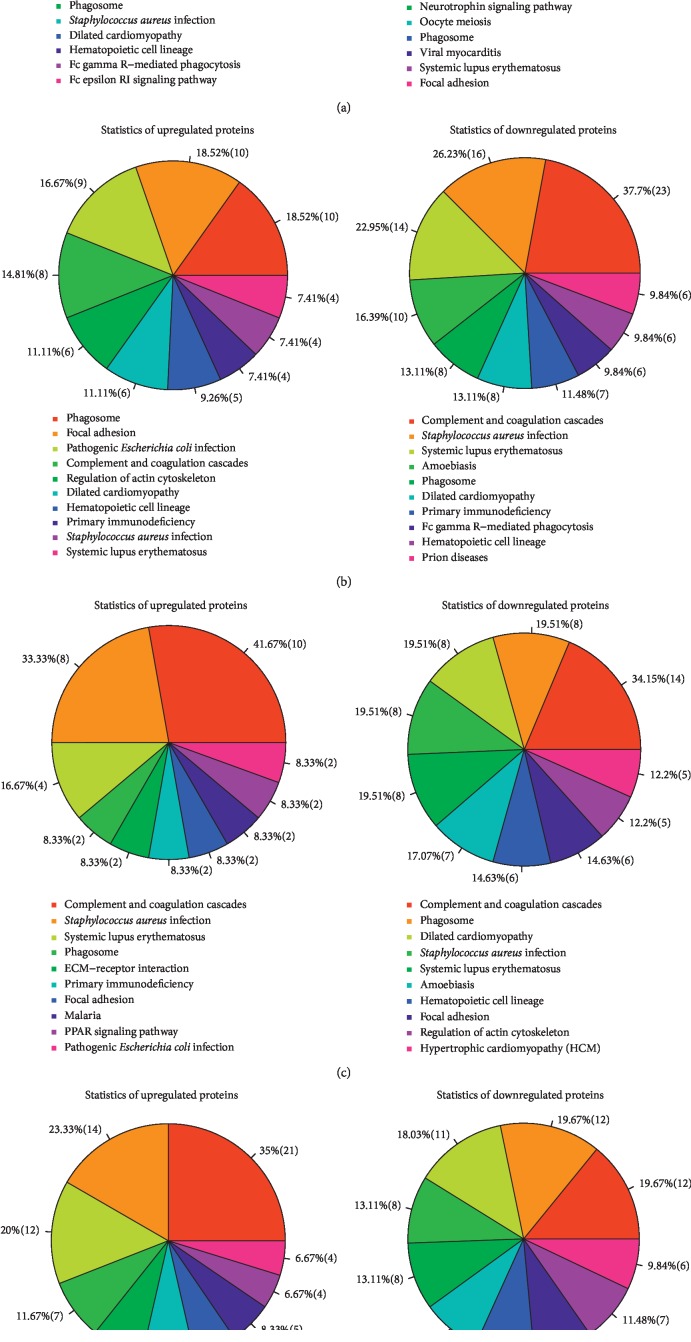
KEGG analysis of the top 10 upregulated and downregulated pathways in (a) MG patients without treatment (T1) vs. healthy volunteers (C); (b) the group of MG patients with routine western medical treatment (T2) vs. MG patients without treatment (T1); (c) MG patients with combined treatments of Jia Wei BZYQ decoction and routine western medicines (T3) vs. MG patients without treatment (T1); (d) the group of MG patients with combined treatments of Jia Wei BZYQ decoction and routine western medicines (T3) vs. MG patients with routine western medical treatment (T2).

Due to the limitations of annotation databases, the number of proteins with annotation information was disparate in different databases. As presented in Figure 1, 567, 216, and 432 annotated proteins were available for GO, COG, and KEGG functional annotation databases, respectively. GO analysis results showed that the pathogenesis of MG was closely related to abnormal immune responses (data not presented). In immune responses, C-reactive protein (CRP) ranked in first place among downregulated proteins in T1 vs. C group, while it was the most upregulated protein in T2 vs. T1 and T3 vs. T1 groups. KEGG analysis on the top 10 upregulated and downregulated pathways disclosed that pathogen (e.g., *Escherichia coli* and *Staphylococcus aureus*) infection-related pathways occupied a larger percentage in T1 vs. C, T2 vs. T1, T3 vs. T1, and T3 vs. T2 groups (Figures 2(a)–2(d)). In T1 vs. C group, the most upregulated protein in Complement and coagulation cascade pathway was plasma kallikrein (KLKB1), while Immunoglobulin heavy variable 6-1 (IGHV6-1) was the most upregulated protein in the pathways of Systemic lupus erythematosus, primary immunodeficiency, amoebiasis, phagosome, *Staphylococcus aureus* infection, Fc gamma R-mediated phagocytosis, Fc epsilon RI signaling pathway, Dilated cardiomyopathy, and Hematopoietic cell lineage. In T1 vs. C group, the most downregulated protein was keratin, type I cytoskeletal 16 (KRT16) in Pathogenic *Escherichia coli* infection and *Staphylococcus aureus* infection pathways with 14-3-3 protein epsilon (YWHAE) in Cell cycle, Neurotrophin signaling pathway, and Oocyte meiosis pathway, von Willebrand factor (VWF) in Focal adhesion pathway, and Immunoglobulin heavy variable 2-26 (IGHV2-26) in the pathways of Phagosome, Viral myocarditis, and Systemic lupus erythematosus. In T2 vs. T1 group, the most upregulated protein was tubulin beta chain (TUBB) in Phagosome and Pathogenic *Escherichia coli* infection pathways with VWF in Focal adhesion pathway, vitamin K-dependent protein C in Complement and coagulation cascades, cofilin-1 in Regulation of actin cytoskeleton pathway, tropomyosin alpha-4 chain in Dilated cardiomyopathy, integrin alpha-IIb (ITGA2B) in Hematopoietic cell lineage pathway, Ig gamma-4 chain C region (IGHG4) in Primary immunodeficiency pathway, haptoglobin in *Staphylococcus aureus* infection pathway, and alpha-actinin-1 in Systemic lupus erythematosus pathway. In T2 vs. T1 group, the most downregulated protein was complement component C9 in Complement and coagulation cascades, Systemic lupus erythematosus, Prion diseases, and Amoebiasis pathways with complement C1q subcomponent subunit C (C1QC) in *Staphylococcus aureus* infection pathway, Ig gamma-1 chain C region (IGHG1) in pathways of Phagosome, Dilated cardiomyopathy, Primary immunodeficiency, Fc gamma R-mediated phagocytosis, and Hematopoietic cell lineage. In T3 vs. T1 group, the most upregulated protein was vitamin K-dependent protein C in the pathway of Complement and coagulation cascades with KRT16 in *Staphylococcus aureus* infection and Pathogenic *Escherichia coli* infection pathways, histone H4 (HIST1H4A) in Systemic lupus erythematosus pathway, IGHG4 in Phagosome and Primary immunodeficiency pathways, VWF in Focal adhesion pathway, hemoglobin subunit alpha (HBA1) in Malaria pathway, and apolipoprotein C-III (APOC3) in PPAR signaling pathway. In T3 vs. T1 group, the most downregulated protein was complement C4-A (C4A) in the pathways of Complement and coagulation cascades, *Staphylococcus aureus* infection, and Systemic lupus erythematosus with thrombospondin-1 (THBS1) in Phagosome and Focal adhesion pathways, tropomyosin alpha-4 (TPM4) in Dilated cardiomyopathy and Hypertrophic cardiomyopathy pathways, vinculin (VCL) in Amoebiasis and Regulation of actin cytoskeleton pathways, and immunoglobulin heavy variable 6-1 (IGHV6-1) in Hematopoietic cell lineage. In T3 vs. T2 group, the most upregulated protein is C9 in the pathways of Complement and coagulation cascades, Amoebiasis, Prion diseases, and Systemic lupus erythematosus, with complement factor H-related protein 1 (CFHR1) in *Staphylococcus aureus* infection pathway, complement C1r subcomponent (C1R) in Phagosome pathway, Ig alpha-2 chain C region (IGHA2) in Primary immunodeficiency, Dilated cardiomyopathy, and Fc gamma R-mediated phagocytosis pathways, and phospholipid transfer protein (PLTP) in PPAR signaling pathway. The most downregulated protein is THBS1 in Phagosome and Focal adhesion pathways, with C4A in Complement and coagulation cascades and *Staphylococcus aureus* infection pathways, ITGA2B in Hematopoietic cell lineage pathway, alpha-actinin-1 (ACTN1) in Systemic lupus erythematosus pathway, myosin-9 (MYH9) in Regulation of actin cytoskeleton pathway, TPM4 in Dilated cardiomyopathy pathway, tubulin alpha-4A chain (TUBA4A) in Pathogenic *Escherichia coli* infection pathway, and glyceraldehyde-3-phosphate dehydrogenase (GAPDH) in Metabolic pathway.

### 3.3. The Identification of Key Proteins by Protein Association Network Analyses

**Figure 3:**
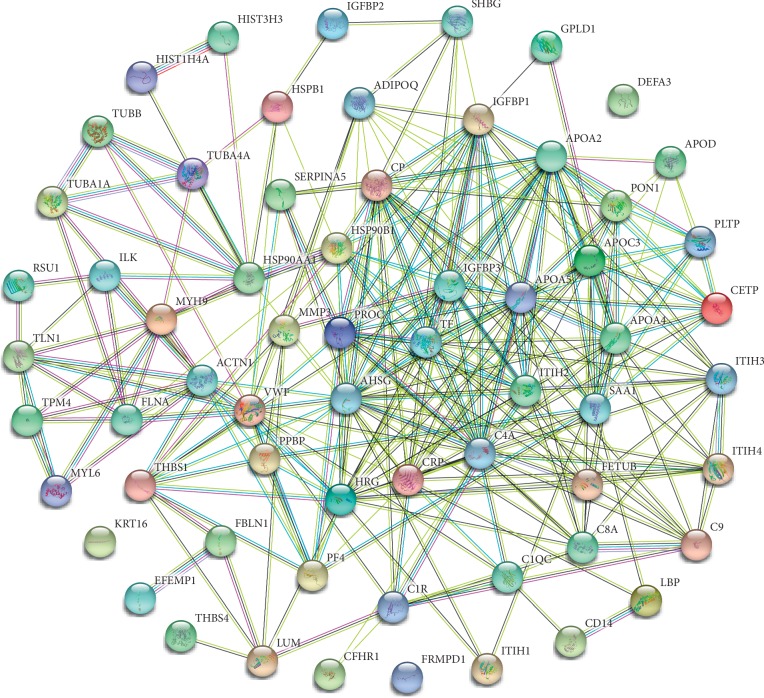
The association network of proteins.

Additionally, Toll-like receptor (TLR), p53 signaling, nucleotide oligomerization domain- (NOD-) like receptor, mitogen-activated protein kinase (MAPK), peroxisome proliferator-activated receptor (PPAR), and transforming growth factor-beta (TGF-beta) signaling pathways have been identified as essential players in immunity and host defense against pathogen infections. In this study, we selected these immune pathway-related proteins and the top 10 upregulated or downregulated proteins in each group, which were integrated and are listed in [Supplementary-material supplementary-material-1]. The association network of proteins in [Supplementary-material supplementary-material-1] was further established via the STRING database by assessing the possibility of protein-protein interactions based on the combined score (Figure 3). The interacted proteins with a combined score ≥0.9 are presented in Table 4. Moreover, we further analyzed the number of proteins that could interact with a random protein (Table 5) to identify key proteins in the protein association network. Results suggested that some proteins (e.g., alpha-2-HS-glycoprotein (AHSG), apolipoprotein A-II (APOA2), apolipoprotein A-IV (APOA4), CRP, ceruloplasmin (CP), complement C4-A (C4A), histidine-rich glycoprotein (HRG), apolipoprotein A-V (APOA5), von Willebrand factor (VWF), apolipoprotein C-III (APOC3), insulin-like growth factor-binding protein 1 (IGFBP1), inter-alpha-trypsin inhibitor heavy chain H2 (ITIH2), vitamin K-dependent protein C (PROC), insulin-like growth factor-binding protein 3 (IGFBP3), endoplasmin (HSP90B1), and serotransferrin (TF)) might play vital roles in MG progression (Table 5). Data for these proteins were collected and are summarized in Table 6 and [Supplementary-material supplementary-material-1].

Data in Table 6 suggested that some proteins such as CRP, TF, APOC3, and VWF might inhibit MG development, while others (e.g., APOA2 and ITIH2) have potential promotional effects on MG progression. Furthermore, our results indicated that routine western medicines alone or along with Jia Wei BZYQ decoction might alleviate MG by regulating the targets in Table 6. The routine treatment plus Jia Wei BZYQ decoction group might experience stronger therapeutic effects due to the upregulation of proteins such as CRP compared with routine treatment group (Table 6). Additionally, our data suggested a link between therapeutic inefficiency and these genes (Table 6). Furthermore, proteomic analyses showed that combined treatment of Jia Wei BZYQ decoction and routine western medicines resulted in abnormal expression of some proteins including myosin heavy chain 9 (MYH9), filamin A (FLNA), tubulin alpha-4A (TUBA4A), thrombospondin-1 (THBS1), tropomyosin alpha-4 (TPM4), Ras suppressor protein 1 (RSU1), ACTN1, and PPBP compared with healthy control group, untreated group, and routine treatment group, indicating that Jia Wei BZYQ decoction might exert its therapeutic effects for MG by regulating these proteins (Table 7).

We also found that monocyte differentiation antigen CD14 expression, related to TLR pathway, was markedly upregulated in T1 vs. C group (ratio: 2.662247514), but was notably downregulated in T2 vs. T1 (ratio: 0.306661468) and T3 vs. T1 (ratio: 0.438830803) groups. Furthermore, TLR pathway-related lipopolysaccharide-binding protein (LBP) expression was noticeably reduced in T1 vs. C group (ratio: 0.102162533) but was remarkably increased in T2 vs. T1 group (ratio: 5.087579717).

## 4. Discussion

MG is both clinically and pathologically heterogeneous disease with multiple targets, treatment responses, and clinical manifestations [42, 43]. Recently, immunotherapy has emerged as an effective approach to improve clinical outcomes for MG patients [44, 45]. Inflammation and immunity-related pathways such as TLRs have been found to be implicated in the pathogenesis of MG [46, 47]. Previous studies showed that BZYQ decoction could reduce side effects of chemotherapeutic drugs and enhance their therapeutic efficiency [48, 49] and improve immune responses by regulating inflammation and immunity-related pathways and molecules [50–52]. For instance, BZYQ decoction inhibited T helper 2 (Th2) responses and promoted interleukin 12 (IL-12) release from macrophages by increasing TLR4 expression in murine allergic rhinitis models [53]. Our study demonstrated that CD14 was abnormally upregulated in MG patients, and routine western medicines alone or along with Jia Wei BZYQ decoction may treat MG by reducing CD14 expression. Prior studies showed that CD14 induced NF-kappa-B activation, cytokine secretion, and immune and inflammatory response by activating TLR4 signaling and TLR signaling cascade [54, 55], suggesting that BZYQ decoction might treat MG by inhibiting CD14/TLR4 signaling pathway. Furthermore, differences in LBP expression suggested that pyridostigmine and/or prednisone might exert therapeutic effects by upregulating LBP expression in MG cases. MG is an autoimmune disease. Pathogenic pathways such as *Staphylococcal aureus* infection, Complement and coagulation cascades, Fc gamma R-mediated phagocytosis, Fc epsilon RI signaling pathway, and some inflammatory factors are usually upregulated in MG. Hence, some proteins among these pathogenic pathways were highly expressed in T1 vs. C group. Prednisone could suppress abnormal immunity responses in MG patients, so the expression of these pathological pathway-related proteins was downregulated in T2 vs. T1 group. The upregulation of these pathological pathway-related proteins in T3 vs. T2 group might result from the inhibitory effect of Jia Wei BZYQ decoction on prednisone-mediated anti-immunity responses. On the other hand, the network of cellular signal regulatory pathways is very complex and above pathological pathway-related proteins may participate in the regulation of multiple pathways with increased expression in one pathway and reduced or unchanged expression in other ways. In addition, the sample number is relatively small in our study and it is imperative to investigate the roles of identified proteins in MG progression through other experiments.

The interaction of proteins plays important roles in coordinating biological behaviors of organisms. KEGG analysis is an effective approach to identify proteins implicated in vital metabolism and signaling transduction pathways. Regarding the vital roles of immune responses in MG etiology and Jia Wei BZYQ decoction treatment, we selected proteins implicated in immune pathways (e.g., PPAR, MAPK, p53, TGF-beta, Wnt, NOD-like receptor, and TLR) by KEGG function annotation analysis in T1 vs. C, T2 vs. T1, T3 vs. T1, and T3 vs. T2 groups. Moreover, the top 10 upregulated and downregulated proteins in T1 vs. C, T2 vs. T1, T3 vs. T1, and T3 vs. T2 groups were picked out, which are presented in [Supplementary-material supplementary-material-1]. CRP, a member of the pentraxin superfamily and a highly conserved acute-phase plasma protein in humans, has been recognized as a regulator of inflammation and autoimmunity [56–58]. In addition, CRP has been found to be implicated in the pathogenesis of multiple diseases such as inflammatory diseases, cardiovascular disease, and cancers [57, 59]. Moreover, prior studies showed that steroid therapy before thymectomy resulted in a marked reduction in serum CRP concentration in MG patients compared with the nonsteroid treatment group [60]. Our data showed that CRP expression was markedly downregulated in T1 vs. C group, but was notably upregulated in T2 vs. T1 and T3 vs. T1 groups, hinting that CRP might inhibit the progression of MG and Jia Wei BZYQ decoction might enhance the therapeutic effect of routine western medicines for MG cases by increasing CRP expression. Additionally, our data indicated that proteins such as TF and VWF might hinder MG development, whereas proteins such as APOA2, ITIH2, and CP might promote MG progression. Proteomic analyses also suggested that Jia Wei BZYQ decoction might exert therapeutic effects for MG by regulating some vital protein expression. Among the proteins we detected, antigen uptake- and presentation-related gene THBS1 has been reported to be highly expressed in thymus tissues of MG patients [61].

Collectively, our data identified some potential biomarkers to facilitate the development of MG diagnosis and treatment. Moreover, our data provided insight into the therapeutic mechanisms of Jia Wei BZYQ decoction and routine western medicines for MG, deepening our understanding of MG pathogenesis. Although our study elucidated potential roles of some proteins in the etiology and pharmacopathology of MG, further *in vitro* and *in vivo* experiments are necessary to confirm our results of the iTRAQ-based plasma proteomics analysis in MG. Additionally, the sample size in our study was small, which is a limitation that should be considered with our results.

## 5. Conclusions

The treatments of Jia Wei BZYQ decoction and routine western medicines resulted in many protein alterations in the plasma of MG patients. Our data presented a valuable resource for diagnosis and treatment for MG.

## Figures and Tables

**Table 1:** Basic clinical parameters of healthy volunteers and MG patients before treatment.

Group	Number	Age of admission	Sex	Disease duration (years)	Neostigmine test	RNS	Thymus pathology	Thymectomy
C	1	53	M	—	ND	ND	Nor	No
	2	69	F	—	ND	ND	Nor	No
	3	49	M	—	ND	ND	Nor	No
T1	4	54	M	1	+	+	Nor	No
	5	49	F	0.2	+	+	Nor	No
	6	44	F	10	+	+	Multiple nodules	No
T2	7	58	F	0.1	+	+	Thymoma	Yes
	8	42	F	27	+	+	Nor	No
	9	43	F	1	+	+	Nor	No
T3	10	58	F	0.1	+	+	Thymoma	Yes
	11	44	F	1	+	+	Thymoma	Yes
	12	52	M	0.6	+	+	Nor	No

Note: F = female; M = male; nor = normal; ND = no detected; “+” = positive; “−” = negative; RNS = repetitive nerve stimulation (a variant of the nerve conduction study where electrical stimulation is delivered to a motor nerve repeatedly several times per second).

**Table 2:** Clinical features of MG patients before and after treatment, related treatment methods, and clinical outcomes.

Group	Number	Maximum severity of disease	AchR Ab (nmol/L)	Titin Ab (nmol/L)	Osserman classification	Outcome	Treatment
		Before/after	Before/after	Before/after	Before/after		
T1	1	Right blepharoptosis/unchanged	—/—	—/—	I/I	SD	—
	2	Limb weakness, blepharoptosis with blurred vision, weak chewing, dysphagia/unchanged	1.8/4.5	—/—	IIA/IIA	PD	—
	3	Right blepharoptosis with double vision/unchanged	8.5/7.3	—/—	I/I	SD	—

T2	4	Drooping eyes with squint, limb weakness/squint, weak limbs when tired	12.3/9.3	—/—	IIA/IIA	PR	PRE, PYR
	5	Drooping eyelids, limb weakness, mild dysphagia/general fatigue, remaining symptoms disappear	16.3/11.7	—/—	IIA/IIA	PR	PRE, PYR
	6	Blepharoptosis with double vision, limb weakness, mild mastication weakness/double vision, remaining symptoms disappear	—/—	—/—	IIA/I	PR	PRE

T3	7	Blepharoptosis with strabismus and weakness of limbs/strabismus, remaining symptoms disappear	12.3/8.4	—/—	IIA/I	PR	PRE, PYR, and Jia Wei BZYQ decoction
	8	Weakness of limbs, blepharoptosis, mild masticatory weakness, dizziness/eyelids lift weakly when excessive use of the eyes, remaining symptoms disappear	11.3/8.9	8.7/1.9	IIA/I	PR	PYR and Jia Wei BZYQ decoction
	9	Double blepharoptosis, double vision/symptoms disappear	3.6/1.3	3.4/2.2	I/I	PR	PYR and Jia Wei BZYQ decoction

Note: AchR Ab normal ranges: <0.4 nmol/L; Titin Ab normal ranges: < 1.0 nmol/L; “−” = negative; SD = stable disease; PD = progression disease; PR = partial remission; PRE = prednisone; PYR = pyridostigmine.

**Table 3:** Protein number analysis of different treatment groups.

Type	T1 vs. C	T2 vs. C	T2 vs. T1	T3 vs. C	T3 vs. T1	T3 vs. T2
The number of quantified proteins	447	447	447	447	447	447
The number of upregulated proteins	67	53	67	64	38	82
The number of downregulated proteins	43	64	76	51	48	76
The number of differently expressed proteins	110	117	143	115	86	158

**Table 4:** Interacted proteins with combined score ≥0.9.

Node1	Node2	Homology	Coexpression	Experimentally_determined_interaction	Database_annotated	Automated_textmining	Combined_score
MYL6	MYH9	0	0.64	0.858	0.9	0.711	0.998
APOA2	APOC3	0	0.796	0	0.9	0.86	0.996
TUBB	TUBA1A	0.92	0.227	0.948	0.9	0.591	0.995
AHSG	APOA2	0	0.918	0	0.9	0.455	0.995
APOA5	APOC3	0	0.466	0	0.9	0.86	0.991
TUBB	TUBA4A	0.92	0.189	0.881	0.9	0.679	0.99
APOA4	APOC3	0	0.565	0	0.9	0.789	0.99
AHSG	ITIH2	0	0.841	0	0.9	0.439	0.99
AHSG	HRG	0	0.67	0	0.9	0.644	0.987
AHSG	TF	0	0.68	0	0.9	0.571	0.985
AHSG	IGFBP1	0	0.788	0	0.9	0.25	0.982
TF	APOA2	0	0.675	0	0.9	0.502	0.982
TUBA1A	TUBA4A	0.986	0	0.809	0.9	0.58	0.98
CD14	LBP	0	0.063	0.379	0.9	0.712	0.98
ILK	RSU1	0	0.265	0.925	0	0.674	0.98
PF4	PPBP	0.886	0.786	0	0.9	0.922	0.98
VWF	THBS1	0	0.063	0	0.9	0.785	0.978
C1R	C4A	0	0.183	0.05	0.9	0.746	0.977
AHSG	PROC	0	0.742	0	0.9	0.128	0.975
APOA2	ITIH2	0	0.728	0	0.9	0.128	0.974
THBS1	HRG	0	0	0.379	0.9	0.609	0.973
SERPINA5	PROC	0	0.177	0.435	0.9	0.416	0.969
APOA5	APOA2	0	0.399	0	0.9	0.526	0.969
C1R	C1QC	0	0.119	0	0.9	0.666	0.968
PF4	VWF	0	0.061	0	0.9	0.685	0.967
TF	ITIH2	0	0.578	0	0.9	0.297	0.967
HIST1H4A	HIST3H3	0	0.107	0.494	0.9	0.371	0.967
TF	CP	0	0.181	0	0.9	0.636	0.967
PPBP	VWF	0	0.061	0	0.9	0.669	0.966
MYL6	TPM4	0	0.185	0.185	0.9	0.546	0.965
APOA2	IGFBP1	0	0.645	0	0.9	0.101	0.965
ACTN1	TLN1	0	0.116	0.083	0.9	0.622	0.965
APOA2	APOA4	0	0.115	0	0.9	0.626	0.964
AHSG	CP	0	0.157	0	0.9	0.609	0.964
ILK	HSP90AA1	0	0	0.161	0.9	0.584	0.962
C8A	C9	0.781	0.341	0.379	0.9	0.576	0.96
APOA5	AHSG	0	0.556	0	0.9	0.189	0.96
C4A	C1QC	0	0.16	0	0.9	0.551	0.959
ITIH2	PROC	0	0.593	0	0.9	0.05	0.957
C4A	APOA2	0	0.083	0.379	0.9	0.308	0.955
PF4	THBS1	0	0.06	0	0.9	0.561	0.955
ITIH2	IGFBP1	0	0.554	0	0.9	0	0.953
ADIPOQ	CRP	0	0	0	0	0.952	0.952
TUBB	HSP90AA1	0	0.129	0.13	0.9	0.411	0.949
HSP90AA1	TUBA1A	0	0.062	0.305	0.9	0.305	0.948
APOA2	PON1	0	0.141	0	0.72	0.794	0.946
APOA2	CP	0	0.115	0	0.9	0.44	0.946
MYL6	TLN1	0	0.427	0	0.9	0.135	0.946
APOA5	TF	0	0.175	0	0.9	0.406	0.946
C4A	TF	0	0.159	0	0.9	0.405	0.945
PF4	HRG	0	0	0	0.9	0.471	0.944
ILK	ACTN1	0	0.064	0.133	0.9	0.38	0.943
PF4	PROC	0	0	0.379	0.9	0.164	0.943
TF	IGFBP1	0	0.214	0.313	0.9	0.073	0.943
CP	PROC	0	0.111	0.379	0.9	0.09	0.943
PLTP	APOA2	0	0	0.379	0.72	0.7	0.943
APOA5	IGFBP1	0	0.394	0	0.9	0.126	0.942
ITIH2	CP	0	0.24	0	0.9	0.289	0.941
APOA5	ITIH2	0	0.428	0	0.9	0	0.94
C4A	CP	0	0.159	0	0.9	0.35	0.94
FLNA	VWF	0	0.061	0.085	0.9	0.38	0.939
ITIH2	FETUB	0	0.916	0	0	0.31	0.939
C1R	CRP	0	0.061	0	0.9	0.399	0.938
APOC3	CETP	0	0.06	0	0.72	0.785	0.938
APOA5	C4A	0	0.106	0	0.9	0.363	0.938
C4A	AHSG	0	0.148	0	0.9	0.322	0.937
APOA5	APOA4	0.681	0.197	0	0.9	0.802	0.937
IGFBP3	IGFBP1	0.755	0.214	0	0.9	0.929	0.936
VWF	PROC	0	0	0	0.9	0.392	0.936
PPBP	THBS1	0	0.062	0	0.9	0.363	0.935
EFEMP1	FBLN1	0.72	0.16	0.12	0.9	0.709	0.935
TF	IGFBP3	0	0.096	0.313	0.9	0.057	0.933
HSP90AA1	TUBA4A	0	0.055	0	0.9	0.342	0.932
C4A	ITIH2	0	0.177	0	0.9	0.237	0.931
APOA5	PROC	0	0.338	0	0.9	0	0.931
SERPINA5	VWF	0	0	0	0.9	0.31	0.928
C4A	PROC	0	0.128	0.05	0.9	0.231	0.927
VWF	HRG	0	0.06	0	0.9	0.292	0.927
TLN1	VWF	0	0.055	0	0.9	0.282	0.926
APOA2	PROC	0	0.275	0	0.9	0.062	0.926
IGFBP1	PROC	0	0.287	0	0.9	0	0.925
AHSG	IGFBP3	0	0.152	0	0.9	0.197	0.925
TPM4	TLN1	0	0.089	0	0.9	0.247	0.925
APOA5	CETP	0	0.061	0	0.72	0.735	0.924
APOA2	CETP	0	0	0	0.72	0.738	0.923
IGFBP1	CP	0	0.146	0	0.9	0.164	0.922
C4A	C8A	0	0.127	0	0	0.914	0.922
PPBP	HRG	0	0	0	0.9	0.25	0.921
ITIH3	ITIH4	0.896	0.18	0	0.9	0.718	0.92
ACTN1	THBS1	0	0.098	0	0.9	0.186	0.92
C1QC	CRP	0	0	0	0.9	0.236	0.92
SAA1	PF4	0	0.061	0	0.9	0.195	0.917
SAA1	PPBP	0	0.063	0	0.9	0.195	0.917
ACTN1	VWF	0	0.061	0.17	0.9	0.066	0.917
IGFBP3	APOA2	0	0.141	0	0.9	0.107	0.916
AHSG	THBS1	0	0	0	0.9	0.189	0.915
TF	HSP90B1	0	0	0	0.9	0.168	0.913
TF	PROC	0	0.147	0.067	0.9	0	0.913
C4A	IGFBP1	0	0.116	0	0.9	0.096	0.913
PON1	CETP	0	0	0	0.72	0.703	0.913
AHSG	PF4	0	0	0	0.9	0.161	0.912
AHSG	VWF	0	0.057	0	0.9	0.144	0.912
APOA5	CP	0	0.116	0	0.9	0.071	0.91
APOA2	HSP90B1	0	0	0	0.9	0.135	0.909
IGFBP3	ITIH2	0	0.112	0	0.9	0	0.907
C4A	IGFBP3	0	0.095	0	0.9	0.061	0.907
AHSG	PPBP	0	0	0	0.9	0.111	0.907
HSP90B1	CP	0	0.077	0	0.9	0.08	0.907
IGFBP3	CP	0	0.076	0	0.9	0.076	0.907
ACTN1	PF4	0	0	0	0.9	0.116	0.907
ACTN1	PPBP	0	0	0	0.9	0.111	0.907
ITIH2	HSP90B1	0	0	0	0.9	0.104	0.906
IGFBP3	HSP90B1	0	0	0	0.9	0.093	0.905
APOA5	IGFBP3	0	0.069	0	0.9	0.061	0.904
AHSG	HSP90B1	0	0	0	0.9	0.072	0.903
C4A	HSP90B1	0	0.061	0	0.9	0.042	0.902
IGFBP3	PROC	0	0.062	0	0.9	0	0.902
AHSG	ACTN1	0	0	0	0.9	0.058	0.901
HSP90B1	IGFBP1	0	0	0	0.9	0.058	0.901
HSP90B1	PROC	0	0	0	0.9	0	0.9
APOA5	HSP90B1	0	0	0	0.9	0	0.9
ACTN1	HRG	0	0	0	0.9	0	0.9

**Table 5:** Number of proteins that could interact with a random protein in [Supplementary-material supplementary-material-1].

Protein name	The numbers of interacted protein
Alpha-2-HS-glycoprotein GN = AHSG	26
Apolipoprotein A-II GN = APOA2	21
Apolipoprotein A-IV GN = APOA4	21
C-reactive protein GN = CRP	21
Ceruloplasmin GN = CP	20
Complement C4-A GN = C4A	20
Histidine-rich glycoprotein GN = HRG	20
Apolipoprotein A-V GN = APOA5	19
von Willebrand factor GN = VWF	18
Apolipoprotein C-III GN = APOC3	17
Insulin-like growth factor-binding protein 1 GN = IGFBP1	17
Inter-alpha-trypsin inhibitor heavy chain H2 GN = ITIH2	17
Vitamin K-dependent protein C GN = PROC	17
Insulin-like growth factor-binding protein 3 GN = IGFBP3	16
Endoplasmin GN = HSP90B1	15
Serotransferrin GN = TF	15
Alpha-actinin-1 GN = ACTN1	13
Complement component C8 alpha chain GN = C8A	13
Serum amyloid A-1 protein GN = SAA1	13
Inter-alpha-trypsin inhibitor heavy chain H3 GN = ITIH3	12
Adiponectin GN = ADIPOQ	11
Serum paraoxonase/arylesterase 1 GN = PON1	11
Thrombospondin-1 GN = THBS1	11
Cholesteryl ester transfer protein GN = CETP	10
Complement component C9 GN = C9	10
Fetuin-B GN = FETUB	10
Heat-shock protein HSP 90-alpha GN = HSP90AA1	10
Complement C1r subcomponent GN = C1R	9
Inter-alpha-trypsin inhibitor heavy chain H4 GN = ITIH4	9
Platelet factor 4 GN = PF4	9
Filamin-A GN = FLNA	8
Myosin-9 GN = MYH9	8
Phospholipid transfer protein GN = PLTP	8
Proplatelet basic protein GN = PPBP	8
Stromelysin-1 GN = MMP3	8
Talin-1 GN = TLN1	8
Apolipoprotein D GN = APOD	6
Complement C1q subcomponent subunit C GN = C1QC	6
Lumican GN = LUM	6
Sex hormone-binding globulin GN = SHBG	6
Heat-shock protein beta-1 GN = HSPB1	5
Integrin-linked protein kinase GN = ILK	5
Plasma serine protease inhibitor GN = SERPINA5	5
Tropomyosin alpha-4 chain GN = TPM4	5
Tubulin alpha-1A chain GN = TUBA1A	5
Tubulin alpha-4A chain GN = TUBA4A	5
Myosin light polypeptide 6 GN = MYL6	4
Tubulin beta chain GN = TUBB	4
Fibulin-1 GN = FBLN1	3
Inter-alpha-trypsin inhibitor heavy chain H1 GN = ITIH1	3
Lipopolysaccharide-binding protein GN = LBP	3
Complement factor H-related protein 1 GN = CFHR1	2
Histone H3.1t GN = HIST3H3	2
Histone H4 GN = HIST1H4A	2
Insulin-like growth factor-binding protein 2 GN = IGFBP2	2
Monocyte differentiation antigen CD14 GN = CD14	2
Phosphatidylinositol-glycan-specific phospholipase D GN = GPLD1	2
Ras suppressor protein 1 GN = RSU1	2
EGF-containing fibulin-like extracellular matrix protein 1 GN = EFEMP1	1
Thrombospondin-4 GN = THBS4	1
FERM and PDZ domain-containing protein 1 GN = FRMPD1	0
Keratin, type I cytoskeletal 16 GN = KRT16	0
Neutrophil defensin 3 GN = DEFA3	0

**Table 6:** Upregulated or downregulated ratio of the top 16 proteins in Table 5 in different groups.

Gene name	T1 vs. C ratio	T2 vs. C ratio	T2 vs. T1 ratio	T3 vs. C ratio	T3 vs. T1 ratio	T3 vs. T2 ratio
C-reactive protein GN = CRP	0.05		9.54		27.77	2.90
Alpha-2-HS-glycoprotein GN = AHSG	1.82	0.13	0.07	1.73		13.16
Serotransferrin GN = TF	0.33		3.23		4.29	
Apolipoprotein C-III GN = APOC3	0.20				3.72	0.46
Apolipoprotein A-II GN = APOA2	4.28		0.12		0.22	1.83
von Willebrand factor GN = VWF	0.26	1.63	6.19	0.66	2.53	0.41
Histidine-rich glycoprotein GN = HRG	4.46		0.54	4.31		1.90
Insulin-like growth factor-binding protein 1 GN = IGFBP1		0.24	0.14		0.61	4.68
Ceruloplasmin GN = CP	2.41	0.13	0.06		0.31	5.59
Apolipoprotein A-IV GN = APOA4	0.37	0.38		1.61	4.42	4.31
Complement C4-A GN = C4A			0.37	0.17	0.13	0.37
Apolipoprotein A-V GN = APOA5				1.86	3.65	1.56
Endoplasmin GN = HSP90B1	2.06				0.66	
Insulin-like growth factor-binding protein 3 GN = IGFBP3		0.28	0.21			5.78
Vitamin K-dependent protein C GN = PROC	0.28	1.88	7.66		4.17	0.53
Inter-alpha-trypsin inhibitor heavy chain H2 GN = ITIH2	10.60		0.28	5.28	0.51	1.80

**Table 7:** Altered proteins in the T3 vs. C, T3 vs. T1, and T3 vs. T2 groups.

Gene name	T3 vs. C ratio	T3 vs. T1 ratio	T3 vs. T2 ratio
Histone H4 GN = HIST1H4A	10.05	9	5.75
Myosin-9 GN = MYH9	0.34	0.27	0.09
Filamin-A GN = FLNA	0.33	0.23	0.07
Alpha-actinin-1 GN = ACTN1	0.27	0.33	0.12
Tubulin alpha-4A chain GN = TUBA4A	0.18	0.26	0.07
Thrombospondin-1 GN = THBS1	0.17	0.08	0.05
Proplatelet basic protein GN = PPBP	0.17	0.12	0.06
Talin-1 GN = TLN1	0.16	0.19	0.07
Tropomyosin alpha-4 chain GN = TPM4	0.11	0.21	0.06
Ras suppressor protein 1 GN = RSU1	0.11	0.16	0.06

## Data Availability

The data used to support the findings of this study are available from the corresponding author upon request.

## Abbreviations

MG: Myasthenia gravis
BZYQ: Bu Zhong Yi Qi
C: Healthy volunteers
T1: MG patients without treatment
T2: MG patients with routine western medical treatment
T3: MG patients with combined treatments of BZYQ decoction and routine western medicines
iTRAQ: Isobaric tags for relative and absolute quantitation
2D-LC-MS/MS: Two-dimensional liquid chromatography-tandem mass spectrometry technologies
MS: Mass spectrometry
TCM: Traditional Chinese medicine
RNS: Repetitive nerve stimulation
TEAB: Triethylammonium bicarbonate
DTT: Dithiothreitol
GO: Gene Ontology
KEGG: Kyoto Encyclopedia of Genes and Genomes
KLKB1: Plasma kallikrein
IGHV6-1: Immunoglobulin heavy variable 6-1
KRT16: Keratin, type I cytoskeletal 16
YWHAE: 14-3-3 Protein epsilon
VWF: von Willebrand factor
IGHV2-26: Immunoglobulin heavy variable 2-26
TUBB: Tubulin beta chain
ITGA2B: Integrin alpha-IIb
IGHG4: Ig gamma-4 chain C region
C1QC: Complement C1q subcomponent subunit C
IGHG1: Ig gamma-1 chain C region
HIST1H4A: Histone H4
HBA1: Hemoglobin subunit alpha
APOC3: Apolipoprotein C-III
C4A: Complement C4-A
THBS1: Thrombospondin-1
TPM4: Tropomyosin alpha-4
VCL: Vinculin
CFHR1: Complement factor H-related protein 1
C1R: Complement C1r subcomponent
IGHA2: Ig alpha-2 chain C region
PLTP: Phospholipid transfer protein
ACTN1: Alpha-actinin-1
MYH9: Myosin-9
TUBA4A: Tubulin alpha-4A chain
GAPDH: Glyceraldehyde-3-phosphate dehydrogenase
COGs: Clusters of orthologous groups of proteins
TLR: Toll-like receptor
NOD: Nucleotide oligomerization domain
MAPK: Mitogen-activated protein kinase
PPAR: Peroxisome proliferator-activated receptor
TGF: Transforming growth factor
CRP: C-reactive protein
TF: Transferrin
AHSG: Alpha-2-HS-glycoprotein
APOA2: Apolipoprotein A-II
ITIH3: Inter-alpha-trypsin inhibitor heavy chain 3
HRG: Histidine-rich glycoprotein
PPBP: Proplatelet basic protein
ITIH4: Inter-alpha-trypsin inhibitor heavy chain family member 4
MYH9: Myosin heavy chain 9
FLNA: Filamin A
RSU1: Ras suppressor protein 1
LBP: Lipopolysaccharide-binding protein
Th2: Inhibited T helper 2
IL-12: Interleukin-12.

## Supplementary Materials

Supplementary Materials All upregulated (ratio ≥ 1.5) and downregulated proteins (ratio ≤0.67) in the groups of T1 vs. C, T2 vs. C, T2 vs. T1, T3 vs. C, T3 vs. T1, T3 vs. T2 are presented in Additional file 1; immune pathway-related proteins and the top 10 upregulated or downregulated proteins in each group were picked out and are presented in Excel S1. The 15 core protein in the protein interaction network (Figure 3) and matching statistical analysis of fold changes in T1 vs. C, T2 vs. C, T2 vs. T1, T3 vs. C, T3 vs. T1, and T3 vs. T2 groups are presented in Excel S2. Protein interaction in Excel S1 was analyzed by STRING database and interacted proteins with combined score >0.4 are presented in Excel S3. Heat map visualization of the differentially expressed proteins in plasma samples of myasthenia gravis (MG) patients (T1) compared with those of the healthy control group (C) is shown in Supplementary Figure 1A. Heat map visualization of the differentially expressed proteins in plasma samples of MG patients with the combined treatment of routine western medicine and BZYQ decoction (T3) compared with those of patients with routine treatment (T2) is shown in Supplementary Figure 1B.
