# Supplementary material for: iTRAQ-Based Proteomics Analysis of Plasma of Myasthenia Gravis Patients Treated with Jia Wei Bu Zhong Yi Qi Decoction
Source: Evid Based Complement Alternat Med. 2019 Dec 13;2019:9147072. doi: 10.1155/2019/9147072 (PMC6930785; doi:10.1155/2019/9147072)
Supplement: Supplementary Materials — All upregulated (ratio ≥ 1.5) and downregulated proteins (ratio ≤0.67) in the groups of T1 vs. C, T2 vs. C, T2 vs. T1, T3 vs. C, T3 vs. T1, T3 vs. T2 are presented in Additional file 1; immune pathway-related proteins and the top 10 upregulated or downregulated proteins in each group were picked out and are presented in Excel S1. The 15 core protein in the protein interaction network (Figure 3) and matching statistical analysis of fold changes in T1 vs. C, T2 vs. C, T2 vs. T1, T3 vs. C, T3 vs. T1, and T3 vs. T2 groups are presented in Excel S2. Protein interaction in Excel S1 was analyzed by STRING database and interacted proteins with combined score >0.4 are presented in Excel S3. Heat map visualization of the differentially expressed proteins in plasma samples of myasthenia gravis (MG) patients (T1) compared with those of the healthy control group (C) is shown in Supplementary Figure 1A. Heat map visualization of the differentially expressed proteins in plasma samples of MG patients with the combined treatment of routine western medicine and BZYQ decoction (T3) compared with those of patients with routine treatment (T2) is shown in Supplementary Figure 1B. [file 9147072.f1.zip › 9147072.f1/Supplementary Figure 1A.pdf]

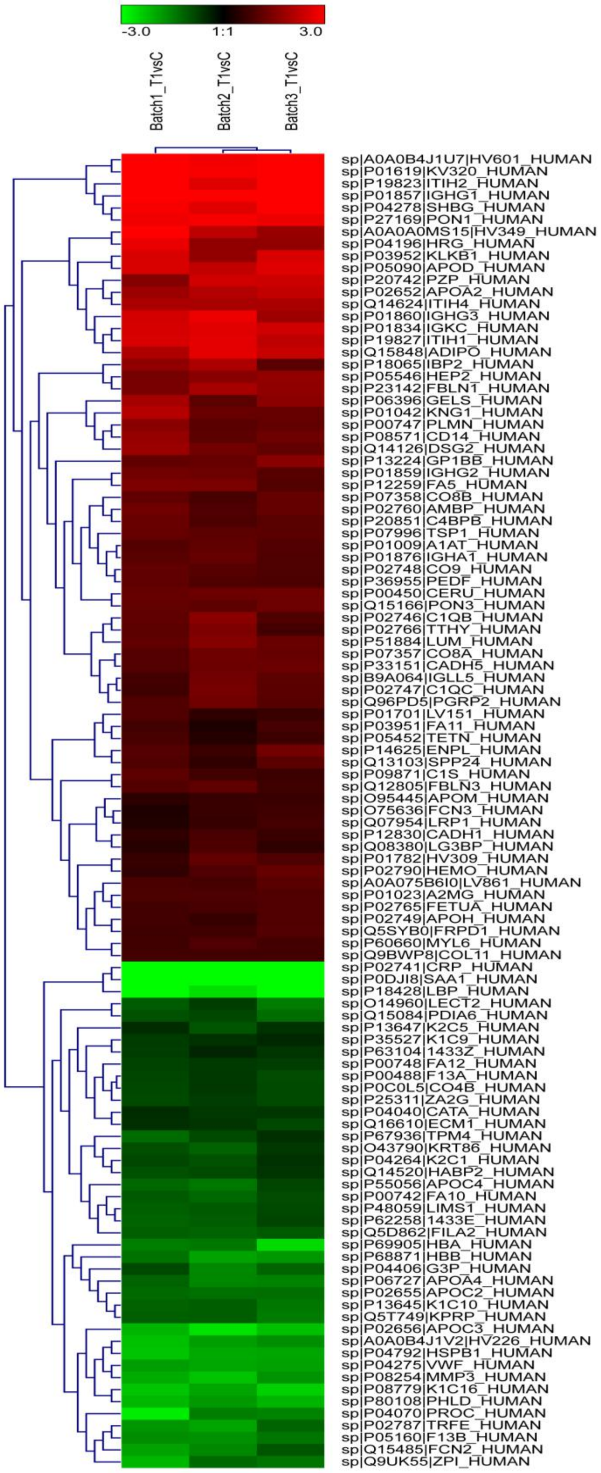

Supplementary Figure 1A: Heat map visualization of the differentially expressed proteins in plasma samples of MG patients (T1) compared with those of the healthy control group (C). Each row represents an individual protein, and each column represents a batch. Green-red heat map values correspond to low-high protein expression. Red represents up-regulation and green represents down-regulation. The gene name (abbreviation) and fold change of the differentially expressed proteins were presented in the T1 vs C excel of Additional file 1 in etail.
