# Supplementary material for: iTRAQ-Based Proteomics Analysis of Plasma of Myasthenia Gravis Patients Treated with Jia Wei Bu Zhong Yi Qi Decoction
Source: Evid Based Complement Alternat Med. 2019 Dec 13;2019:9147072. doi: 10.1155/2019/9147072 (PMC6930785; doi:10.1155/2019/9147072)
Supplement: Supplementary Materials — All upregulated (ratio ≥ 1.5) and downregulated proteins (ratio ≤0.67) in the groups of T1 vs. C, T2 vs. C, T2 vs. T1, T3 vs. C, T3 vs. T1, T3 vs. T2 are presented in Additional file 1; immune pathway-related proteins and the top 10 upregulated or downregulated proteins in each group were picked out and are presented in Excel S1. The 15 core protein in the protein interaction network (Figure 3) and matching statistical analysis of fold changes in T1 vs. C, T2 vs. C, T2 vs. T1, T3 vs. C, T3 vs. T1, and T3 vs. T2 groups are presented in Excel S2. Protein interaction in Excel S1 was analyzed by STRING database and interacted proteins with combined score >0.4 are presented in Excel S3. Heat map visualization of the differentially expressed proteins in plasma samples of myasthenia gravis (MG) patients (T1) compared with those of the healthy control group (C) is shown in Supplementary Figure 1A. Heat map visualization of the differentially expressed proteins in plasma samples of MG patients with the combined treatment of routine western medicine and BZYQ decoction (T3) compared with those of patients with routine treatment (T2) is shown in Supplementary Figure 1B. [file 9147072.f1.zip › 9147072.f1/Supplementary Figure 1B.pdf]

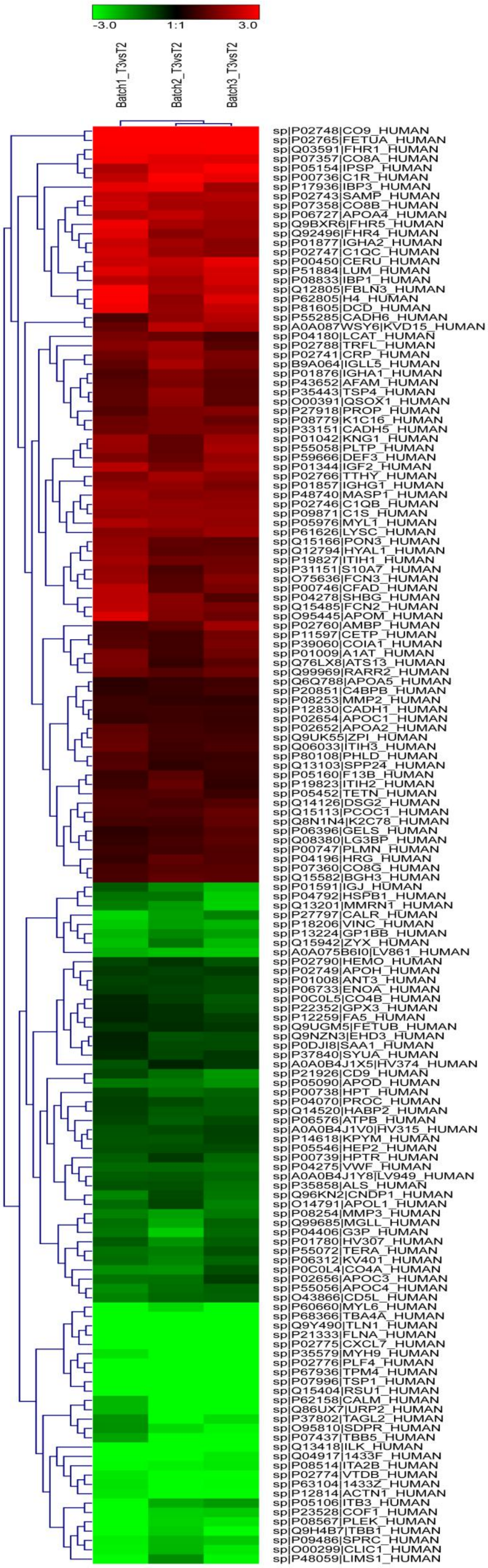

-3.0
1:1
3.0
Batch1_T3vsT2
Batch2_T3vsT2
Batch3_T3vsT2
sp|P02748|CO9_HUMAN
sp|P02765|FETUA_HUMAN
sp|Q03591|FHR1_HUMAN
sp|P07357|CO8A_HUMAN
sp|P05154|IPSP_HUMAN
sp|P00736|C1R_HUMAN
sp|P17936|IBP3_HUMAN
sp|P02743|SAMP_HUMAN
sp|P07358|CO8B_HUMAN
sp|P06727|APOA4_HUMAN
sp|Q9BXR6|FHR5_HUMAN
sp|Q92496|FHR4_HUMAN
sp|P01877|IGHA2_HUMAN
sp|P02747|C1QC_HUMAN
sp|P00450|CERU_HUMAN
sp|P51884|LUM_HUMAN
sp|P08833|IBP1_HUMAN
sp|Q12805|FBLN3_HUMAN
sp|P62805|H4_HUMAN
sp|P81605|DCD_HUMAN
sp|P55285|CADH6_HUMAN
sp|A0A087WSY6|KVD15_HUMAN
sp|P04180|LCAT_HUMAN
sp|P02788|TRFL_HUMAN
sp|P02741|CRP_HUMAN
sp|B9A064|IGLL5_HUMAN
sp|P01876|IGHA1_HUMAN
sp|P43652|AFAM_HUMAN
sp|P35443|TSP4_HUMAN
sp|O00391|QSOX1_HUMAN
sp|P27918|PROP_HUMAN
sp|P08779|K1C16_HUMAN
sp|P33151|CADH5_HUMAN
sp|P01042|KNG1_HUMAN
sp|P55058|PLTP_HUMAN
sp|P59666|DEF3_HUMAN
sp|P01344|IGF2_HUMAN
sp|P02766|TTHY_HUMAN
sp|P01857|IGHG1_HUMAN
sp|P48740|MASP1_HUMAN
sp|P02746|C1QB_HUMAN
sp|P09871|C1S_HUMAN
sp|P05976|MYL1_HUMAN
sp|P61626|LYSC_HUMAN
sp|Q15166|PON3_HUMAN
sp|Q12794|HYAL1_HUMAN
sp|P19827|ITIH1_HUMAN
sp|P31151|S10A7_HUMAN
sp|O75636|FCN3_HUMAN
sp|P00746|CFAD_HUMAN
sp|P04278|SHBG_HUMAN
sp|Q15485|FCN2_HUMAN
sp|O95445|APOM_HUMAN
sp|P02760|AMBP_HUMAN
sp|P11597|CETP_HUMAN
sp|P39060|COIA1_HUMAN
sp|P01009|A1AT_HUMAN
sp|Q76LX8|ATS13_HUMAN
sp|Q99969|RARR2_HUMAN
sp|Q6Q788|APOA5_HUMAN
sp|P20851|C4BPB_HUMAN
sp|P08253|MMP2_HUMAN
sp|P12830|CADH1_HUMAN
sp|P02654|APOC1_HUMAN
sp|P02652|APOA2_HUMAN
sp|Q9UK55|ZPI_HUMAN
sp|Q06033|ITIH3_HUMAN
sp|P80108|PHLD_HUMAN
sp|Q13103|SPP24_HUMAN
sp|P05160|F13B_HUMAN
sp|P19823|ITIH2_HUMAN
sp|P05452|TETN_HUMAN
sp|Q14126|DSG2_HUMAN
sp|Q15113|PCOC1_HUMAN
sp|Q8N1N4|K2C78_HUMAN
sp|P06396|GELS_HUMAN
sp|Q08380|LG3BP_HUMAN
sp|P00747|PLMN_HUMAN
sp|P04196|HRG_HUMAN
sp|P07360|CO8G_HUMAN
sp|Q15582|BGH3_HUMAN
sp|P01591|IGJ_HUMAN
sp|P04792|HSPB1_HUMAN
sp|Q13201|MMRN1_HUMAN
sp|P27797|CALR_HUMAN
sp|P18206|VINC_HUMAN
sp|P13224|GP1BB_HUMAN
sp|Q15942|ZYX_HUMAN
sp|A0A075B6I0|LV861_HUMAN
sp|P02790|HEMO_HUMAN
sp|P02749|APOH_HUMAN
sp|P01008|ANT3_HUMAN
sp|P06733|ENOA_HUMAN
sp|P0C0L5|CO4B_HUMAN
sp|P22352|GPX3_HUMAN
sp|P12259|FA5_HUMAN
sp|Q9UGM5|FETUB_HUMAN
sp|Q9NZN3|EHD3_HUMAN
sp|P0DJI8|SAA1_HUMAN
sp|P37840|SYUA_HUMAN
sp|A0A0B4J1X5|HV374_HUMAN
sp|P21926|CD9_HUMAN
sp|P05090|APOD_HUMAN
sp|P00738|HPT_HUMAN
sp|P04070|PROC_HUMAN
sp|Q14520|HABP2_HUMAN
sp|P06576|ATPB_HUMAN
sp|A0A0B4J1V0|HV315_HUMAN
sp|P14618|KPYM_HUMAN
sp|P05546|HEP2_HUMAN
sp|P00739|HPTR_HUMAN
sp|P04275|VWF_HUMAN
sp|A0A0B4J1Y8|LV949_HUMAN
sp|P35858|ALS_HUMAN
sp|Q96KN2|CNDP1_HUMAN
sp|O14791|APOL1_HUMAN
sp|P08254|MMP3_HUMAN
sp|Q99685|MGLL_HUMAN
sp|P04406|G3P_HUMAN
sp|P01780|HV307_HUMAN
sp|P55072|TERA_HUMAN
sp|P06312|KV401_HUMAN
sp|P0C0L4|CO4A_HUMAN
sp|P02656|APOC3_HUMAN
sp|P55056|APOC4_HUMAN
sp|O43866|CD5L_HUMAN
sp|P60660|MYL6_HUMAN
sp|P68366|TBA4A_HUMAN
sp|Q9Y490|TLN1_HUMAN
sp|P21333|FLNA_HUMAN
sp|P02775|CXCL7_HUMAN
sp|P35579|MYH9_HUMAN
sp|P02776|PLF4_HUMAN
sp|P67936|TPM4_HUMAN
sp|P07996|TSP1_HUMAN
sp|Q15404|RSU1_HUMAN
sp|P62158|CALM_HUMAN
sp|Q86UX7|URP2_HUMAN
sp|P37802|TAGL2_HUMAN
sp|O95810|SDPR_HUMAN
sp|P07437|TBB5_HUMAN
sp|Q13418|ILK_HUMAN
sp|Q04917|1433F_HUMAN
sp|P08514|ITA2B_HUMAN
sp|P02774|VTDB_HUMAN
sp|P63104|1433Z_HUMAN
sp|P12814|ACTN1_HUMAN
sp|P05106|ITB3_HUMAN
sp|P23528|COF1_HUMAN
sp|P08567|PLEK_HUMAN
sp|Q9H4B7|TBB1_HUMAN
sp|P09486|SPRC_HUMAN
sp|O00299|CLIC1_HUMAN
sp|P48059|LIMS1_HUMAN

Supplementary Figure 1B: Heat map visualization of the differentially expressed proteins in plasma samples of MG patients with the combined treatment of routine western medicine and Jia Wei BZYQ decoction (T3) compared with those of patients with routine treatment (T2). Each row represents an individual protein, and each column represents a batch. Green-red heat map values correspond to low-high protein expression. Red represents up-regulation and green represents down-regulation. The gene name (abbreviation) and fold change of the differentially expressed proteins were presented in the T3 vs T2 excel of Additional file 1 in detail.
